# Supplementary material for: Genomic and Proteomic Analysis of Six Vi01-like Phages Reveals Wide Host Range and Multiple Tail Spike Proteins
Source: Viruses. 2024 Feb 13;16(2):289. doi: 10.3390/v16020289 (PMC10892097; doi:10.3390/v16020289)
Supplement: Supplementary file 1 [file viruses-16-00289-s001.zip › Supplementary Table Key.pdf]

Genomic and Proteomic Analysis of SixVi01-like Phages Reveals Wide Host Range and Multiple Tail Spike Proteins

The following supplementary tables are supplied as excel files:

Supplementary Table S1. 150 Vi01-like *Enterobacteriaceae Ackermannviridae.* The 150 phages utilized in this study organized by subcluster and genera, and are listed with a link to NCBI, their GenBank accession number, associated reference, and MCP family (if they share identical major capsid proteins (MCP).

Supplementary Table S2: Use of Structural Homology and Operons to Identify Putative Functions of Vi01-like proteins. Tables are provided for phages FrontPhageNews and ChubbyThor of retrieved structural hits having greater than 60% confidence from Phyre2 analysis of hypothetical proteins. The HHPRED values greater than 50% confidence are given for the same proteins. In addition, predicted tables are provided for predicted operons for each phage.

Supplementary Table S3: Mass spectrometry of Sajous1 virions. Note the gp number conversion due to subsequent rearrangement of bp1 in this genome.

Supplementary Table S4: Strain information for *Salmonella, Shigella,* C*itrobacter* and *E. coli* clinical isolates utilized in this study. Strain information for clinical isolates utilized in this study. *Salmonella*, *Shigella* and *Citrobacter* clinical isolates were obtained from the CDC while E. coli isolates were obtained from Intermountain Healthcare.
